# Phosphoric Acid–Modified TiO_2_ Nanoparticles as Functional Additives for High‐Performance Polyethersulfone Ultrafiltration Membranes

**DOI:** 10.1002/wer.70576

**Published:** 2026-09-10

**Authors:** Assala M. Al‐Jubouri, Kadhum M. Shabeeb, Qusay F. Alsalhy, Saad Al‐Saadi, Adel Zrille, Muayad Al‐shaeli

**Affiliations:** ^1^ Materials Engineering Department University of Technology, Iraq Baghdad Iraq; ^2^ Membrane Technology Research Unit, Chemical Engineering Department University of Technology, Iraq Baghdad Iraq; ^3^ Department of Mechanical and Aerospace Engineering Monash University Clayton Victoria Australia; ^4^ Research Unit Advanced Materials, Applied Mechanics, Innovative Processes and Environment Higher Institute of Applied Sciences and Technology of Gabes (ISSAT), University of Gabes Gabes Tunisia; ^5^ Institute for Sustainability and Innovation Victoria University Melbourne Victoria Australia

**Keywords:** contact angle, fouling, hydrophilicity, permeability, PES membranes, polymeric membranes, porosity, TiO_2_

## Abstract

Polyethersulfone (PES) ultrafiltration membranes were modified with phosphoric acid–functionalized TiO_2_ nanoparticles (0–1 wt%) via phase inversion to enhance permeability, antifouling performance, and dye removal for wastewater treatment. Structural and morphological analyses confirmed the successful modification and uniform incorporation of TiO_2_ within the PES matrix. The membrane containing 0.5 wt% TiO_2_ exhibited optimal performance, achieving the lowest contact angle (~39°), highest porosity (~65.5%), and maximum pure water flux (~292 L·m^−2^·h^−1^), compared to 204 L·m^−2^·h^−1^ for pristine PES. Rhodamine B rejection exceeded 85%, driven by electrostatic interactions between phosphate groups and cationic dye molecules. Fouling tests using bovine serum albumin revealed a high FRR (~92%) and reduced irreversible fouling. Excessive TiO_2_ loading led to nanoparticle agglomeration and reduced permeability.

## Summary

- Phosphoric acid–modified TiO_2_ nanoparticles were successfully synthesized and incorporated into PES membranes.
- Surface functionalization enhanced nanoparticle dispersion and membrane hydrophilicity.
- Modified membranes showed improved permeability and pore structure at optimal TiO_2_ loading, with significant reduction in fouling and irreversible resistance.
- Excess nanoparticle loading caused agglomeration, reducing permeability and antifouling performance.

## Graphical abstract

Phosphoric acid–modified TiO_2_ nanoparticles significantly enhance the performance of polyethersulfone ultrafiltration membranes by improving hydrophilicity, permeability, and antifouling resistance. The surface‐functionalized nanoparticles promote stable dispersion and favorable membrane morphology, resulting in superior separation efficiency and long‐term operational stability.

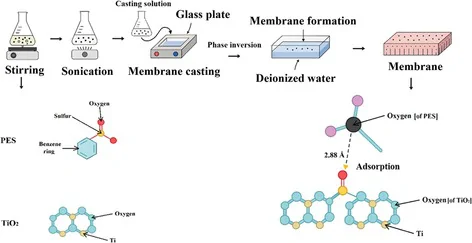

## 1 Introduction

Water is one of the essential resources for the survival of humans, animals, and plants. However, water pollution has become a serious problem threatening the existence of life on earth, mainly due to rapid technological and scientific advances. To address water pollution, scientists and researchers are developing new processes and improving existing water purification methods (Sagasta et al. 2017; Islam et al. 2026). The reuse of water and wastewater, facilitated by modern treatment techniques, is regarded as one of the most effective solutions to the scarcity of clean water sources (Aljumaily et al. 2022; Patel et al. 2025). A wider variety of treatment methods can be utilized, including biological, mechanical, thermal, chemical, and physical methods, or combinations of these, depending on the type and concentration of contaminants in the water (Saravanan et al. 2017; Bhadra et al. 2019; Huo et al. 2026; Zhang et al. 2026). For example, when treating manufacturing wastewater containing dyes and other emulsifying substances, a crucial aspect of wastewater treatment involves physical, biological, and chemical processes designed to remove these contaminants. However, biological treatment methods often face challenges in effectiveness due to the high salinity found in wastewater containing dyes and the complex nature of synthetic pigments. Additionally, methods such as chlorination and ozonation tend to incur high operational costs. As a result, various physical techniques, including adsorption on activated carbon, ultrafiltration, reverse osmosis, and coagulation with chemical agents, have been employed to address this issue. Specifically, ultrafiltration with polymeric membranes has proven highly effective in separating contaminants (Berger and Domingo 2009).

Despite their many advantages, membrane processes are often limited by fouling, which reduces permeance and adversely affects both the quantity and quality of the permeate. Fouling is governed by factors such as feed composition, operating conditions, and membrane surface properties, and although cleaning procedures such as backwashing can partially restore flux, irreversible fouling remains a major challenge. Because membrane surface characteristics strongly influence performance and durability, various chemical and physical modification techniques have been developed to enhance hydrophilicity and reduce fouling (Sharma et al. 2018). These approaches include dehydrochlorination and grafting (Al‐Bahate et al. 2019), as well as dipping, coating, plasma treatment, and blending (Rivera‐Utrilla et al. 2013; Ahmed et al. 2015). Among them, the incorporation of nanoparticles has attracted considerable attention because it enhances the physicochemical properties of membranes, leading to improved antifouling performance and permeability through reduced surface roughness, antimicrobial activity, narrower pore‐size distribution, and increased porosity (Saravanan et al. 2017; Al‐Araji et al. 2022).

Among photocatalytic semiconductors, titanium dioxide (TiO_2_) is widely used for water treatment and water splitting because of its strong oxidizing ability, low toxicity, chemical stability, and low cost (El Sharkawy et al. 2023; El Mchaouri et al. 2025). The addition of TiO_2_ nanoparticles to water filtration membranes has been shown to improve their flux, pollutant removal efficiency, and resistance to fouling (Ayyaru and Ahn 2018; Yu et al. 2024). However, the agglomeration of nanoparticles poses a significant challenge in achieving a uniform surface (Razmjou et al. 2011; Rajaeian et al. 2013; Zhang et al. 2025). Razmjou et al. (2011) studied mechanical and chemical modification approaches using Degussa P25 TiO_2_ nanoparticles to improve dispersion. They found that incorporating modified nanoparticles into PES ultrafiltration membranes significantly improved fouling behavior. For example, the flux recovery improved from around 60% for control and 57% for unmodified nanoparticles to 84% for chemically and mechanically modified particles at a 2 wt% TiO_2_ loading. Hydrophilicity was also improved by around 18%, regardless of the type of modification or the content of TiO_2_ particles in the membrane. In another study, Razmjou et al. (2012) examined the effect of modified TiO_2_ nanoparticles on the polyethersulfone (PES) ultrafiltration hollow fiber membranes. A significant improvement in fouling behavior for chemically and mechanically modified particles at a 2 wt% TiO_2_ loading was observed. The results showed that flux recovery improved by 24%, whereas transmembrane pressure (TMP) was considerably lower during filtration with bovine serum albumin (BSA). After undergoing chemical and mechanical modifications, the surface pore size and overall porosity increased, whereas both surface free energy and roughness decreased.

In the study by Zangeneh et al. (2018), self‐cleaning K‐B‐N triple‐doped‐TiO_2_/PES nanofiltration membranes (0.1–1 wt%) were developed via phase inversion and evaluated for their permeability, antifouling, and post‐treatment performance for biologically treated palm oil mill effluent (POME). The modified membrane with 0.5 wt% exhibited optimal hydrophilicity, flux, and fouling resistance, achieving ~98% color removal and 90% COD removal. To enhance TiO_2_ photocatalytic activity, Sun et al. (2019) modified TiO_2_ with dopamine (TiO_2_@PDA), achieving 99.28% and 77.40% (after 1.5 h) rhodamine B degradation under UV‐Vis and visible light, respectively, with a 35.04% improvement in visible‐light performance over pristine TiO_2_. Similarly, Vatanpour et al. (2022) incorporated a TiO_2_/carbon dots (TiO_2_/CDs) hydrophilic nanocomposite into RO membranes, resulting in smoother, more hydrophilic surfaces, improved antifouling properties, and enhanced photocatalytic self‐cleaning performance, increasing the flux recovery ratio (FRR) from 94.4% to 97.1% after UV‐assisted cleaning.

Despite the considerable progress achieved by incorporating TiO_2_ nanoparticles into polymeric membranes, several challenges remain, particularly nanoparticle agglomeration, limited visible‐light activity, and reduced long‐term stability. Conventional TiO_2_ retains a relatively wide band gap (3.2 eV), limiting its photocatalytic activity to ultraviolet irradiation. Hurum et al. (2003) reported that this limitation reduces the effectiveness of pristine TiO_2_ under natural sunlight. Surface functionalization offers a promising strategy to overcome these drawbacks by improving nanoparticle dispersion and enhancing interfacial interactions within the polymer matrix. In this work, phosphoric acid–modified TiO_2_ nanoparticles were incorporated into PES ultrafiltration membranes via the phase‐inversion method. The phosphate‐functionalized nanoparticles act not only as hydrophilic modifiers but also provide additional electrostatic interaction sites that facilitate the rejection of cationic dye molecules. The effects of nanoparticle loading on membrane morphology, hydrophilicity, porosity, permeability, antifouling behavior, and rhodamine B removal were systematically investigated. The results demonstrate that an optimal loading of 0.5 wt% provides the best balance between water flux, fouling resistance, and dye rejection, highlighting the potential of phosphoric acid‐functionalized TiO_2_ as an effective additive for next‐generation wastewater.

## 2 Materials and Methods

### 2.1 Materials

The materials used in this work included ethyl alcohol (99% purity) and phosphoric acid (H_3_PO_4_) with a minimum assay of 85% and a molar mass of 98 g/mol, both procured from Panreac Applichem, Germany. Titanium oxide nanoparticles/nano powder (TiO_2_) in the range of 10–30 nm were supplied by Skyspring Nanomaterials Inc., USA, with a purity of 99.5%. The PES (Ultrason E6020P) was used, with a molecular weight of 51 kDa. *N*,*N*‐Dimethylacetamide with linear formula: CH_3_CON(CH_3_)_2_, and synonyms: DMAc; with 99.8% purity from Sigma‐Aldrich, Germany; Polyvinylpyrrolidone (PVP) with molecular weight 40 KDa from Central Drug House, India; rhodamine B dye powder form with molecular weight 479.01 g/mol from Sigma‐Aldrich.

### 2.2 Preparation Processes

#### 2.2.1 Titanium Oxide Modification Process

Sol A was prepared by mixing 1 g of TiO_2_ powder with 10 mL of ethanol in a flask, then stirring for 15 min with a magnetic stirrer. Concurrently, in another flask, 1.1764 g of phosphoric acid was mixed with 50 mL of deionized water and stirred for 2 h on a magnetic stirrer to prepare solution B. Once both sol A and solution B were prepared, the next step in the modification process involved combining sol and solution in a single flask and stirring the mixture for 2 h with a magnetic stirrer. After the mixture was homogenized, the resultant was washed three times with deionized water and then dried at 80°C.

After this, the dried product was heated to 450°C at a rate of 5°C/min for 30 min. Nanoparticles were allowed to cool naturally to room temperature for 24 h, resulting in the modified titanium oxide nanoparticles.

##### 2.2.1.1 Modification of the Titanium Oxide Mechanism

**FIGURE 1:**
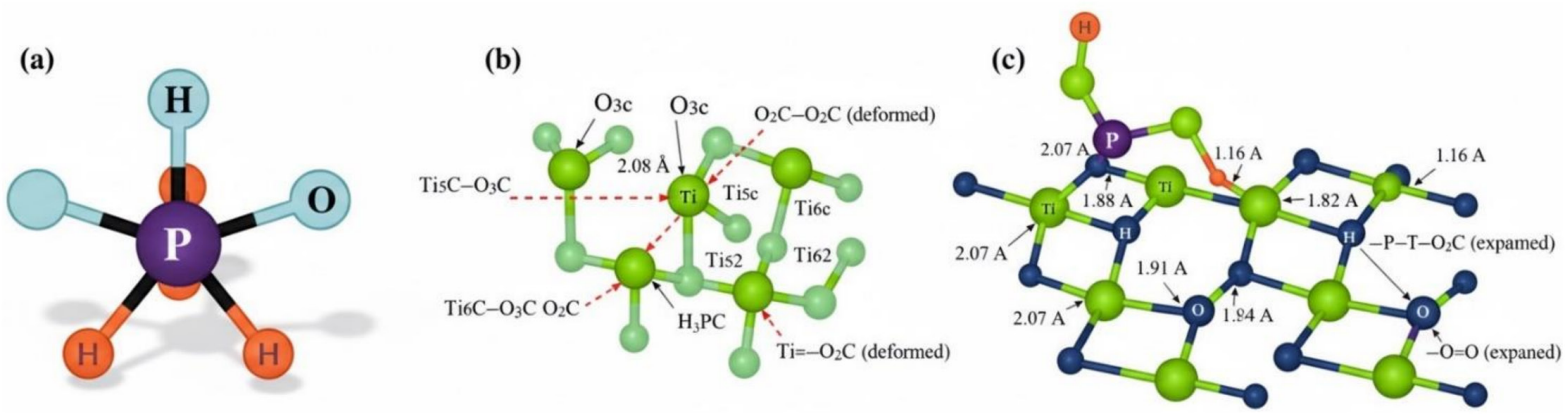
Modification process of titanium oxide: (a) phosphoric acid molecule, (b) surface of anatase (101), and (c) adsorption mechanism of phosphoric acid on anatase surface.

The principle of modifying anatase titanium dioxide (TiO_2_) with phosphoric acid involves the adsorption of H_3_PO_4_ onto the TiO_2_ surface, leading to the formation of −O–Ti_5_C and H–O_2_C bonds. Figure 1 illustrates the adsorption mechanism of phosphoric acid on the anatase surface, leading to a stable geometric configuration. As shown in Figure 1b, the surface of TiO_2_ was consist of 3‐fold coordinated O atoms (O_3C_), 5‐fold coordinated Ti atoms (Ti_5C_), 2‐fold coordinated O atoms (O_2C_), and 6‐fold coordinated Ti atoms (Ti_6c_), and the Ti–O distances (Ti_5C_–O_3C_, Ti_5C_–O_2C_, and Ti_6C_–O_2C_) were 2.08, 1.82, and 1.86 Å, respectively. The P^3^ adsorption configuration forms bonds between H(H_3_PO_4_)‐O_2C_ and =O(H_3_PO_4_)‐Ti_5C_, with lengths of 1.37 and 2.07 Å. Both the phosphoric acid molecule and the surface's lattice structure undergo structural deformation. For example, the bond length of Ti_6C_‐O_2C_ and Ti_5C_‐O_2C_ is greater than before (1.86–1.88 Å and 1.82–1.91 Å). The bonds of O‐H and P=O of phosphoric acid molecules have expanded (0.97–1.09 Å and 1.45–1.49 Å).

Phosphate groups from phosphoric acid that bind to the titanium oxide surface significantly enhance the production of primary reactive radicals, particularly the hydroxyl radical (•OH). This process also increases the reactivity of hydroxyl radicals. The negative electrostatic field created by phosphate anions in the TiO_2_ surface layer, along with the activation of water (H_2_O) through hydrogen bonds formed between phosphate and water molecules, is likely the primary reason for the enhanced photocatalytic activity of modified TiO_2_ (Wang and Liu 2023). Thus, adding phosphoric acid to TiO_2_ enhances its photocatalytic properties by generating more radicals and accelerating the activation of water molecules. However, it also makes it harder for some substrates to adhere to the surface, which can slow degradation via the hole‐oxidation pathway.

#### 2.2.2 Preparation Process of the Membranes

The first step in preparing the pristine membrane by the phase‐inversion method is to dry the polymer at 50°C for 1 h to remove any humidity present in the polymer and then place the dried polymer in a sealed plastic bag to prevent moisture reabsorption. The second step involves the preparation of seven different solutions by mixing the PES with the solvent (DMAc), TiO_2_ nanoparticles in different percentages, and 1% PVP, as shown in the table below. Table 1 presents the details of the chemicals used, along with their corresponding percentages.

**TABLE 1:** The percentages of different materials used to prepare the membranes used in the present study.

Membrane code	Polymer (%)	Solvent (%)	Modified TiO_2_ nanoparticles (%)	PVP (%)
M_0_	15	84	—	1
M_0.2_	15	83.8	0.2	1
M_0.4_	15	83.6	0.4	1
M_0.5_	15	83.5	0.5	1
M_0.6_	15	83.4	0.6	1
M_0.8_	15	83.2	0.8	1
M_1_	15	83.1	1	1

All samples were sonicated by using an Ultrasonic (KQ32OOE) device with an operation frequency of 40 kHz for 30 min to ensure homogeneous dispersion and remove bubbles from the solution. The solution is then cast by using a knife‐casting machine. Then, the cast membrane on a glass substrate was immersed in a water bath at 35°C to initiate coagulation. Finally, the prepared membrane was rinsed and stored in distilled water for 14 days, with the water changed every 2 days, as shown in Figure S1, to ensure complete removal of residual solvent, unreacted additives, and other leachable components.

Figure S1 shows the interaction mechanism between PES and titanium oxide (TiO_2_) nanoparticles during the membrane fabrication, which occurs at the molecular level. In this process, double‐bonded oxygen atoms (O) connect to a sulfur atom (S), forming a sulfonyl group (SO_2_), which is then linked to a benzene ring (an aromatic hydrocarbon) that constitutes the PES molecule. PES bind to or interact with the TiO_2_ surface, which features a repeating lattice that represents the crystalline structure of titanium dioxide, comprising both oxygen atoms (O) and titanium atoms (Ti).

### 2.3 Characterization

#### 2.3.1 X‐Ray Diffraction (XRD)

The structural characterization of modified nanoparticles was performed using X‐ray diffraction (Shimadzu, XRD‐600) to determine the crystalline structure of the compounds. The inspection was performed at the Nanotechnology and Advanced Materials Research Center/University of Technology, Baghdad/Iraq.

#### 2.3.2 Fourier Transform Infrared Spectroscopy (FTIR)

The functional groups of both the neat and the modified nanoparticle membranes were characterized using Fourier transform infrared spectroscopy (FTIR) (TENSOR‐27, Bruker Optics Co., Germany) in the range of 500–4000 cm^−1^. The membranes were analyzed by directly placing small pieces of the membrane on the attenuated total reflectance (ATR) crystal of the FTIR spectrometer. The resulting spectra provided information about the chemical structure and confirmed the successful incorporation of modified nanoparticles or functional moieties into the polymer matrix. All measurements were performed at room temperature in the Department of Materials Engineering, University of Technology, Baghdad, Iraq.

#### 2.3.3 Scanning Electron Microscopy (SEM) and Elemental Analysis (EDX)

A scanning electron microscope (F50 FE‐SEM) operating at an accelerating voltage of 20 kV was used to examine the surface morphologies of the membranes and their cross‐sections. Before analyzing the cross‐sections, the membranes were exposed to liquid nitrogen, which rendered them brittle and facilitated easier fracture. The tests were conducted at Al‐Khora Company in Baghdad, Iraq.

#### 2.3.4 Atomic Force Microscopy (AFM)

The AFM device was used to investigate the three‐dimensional surface roughness and morphology of both neat and TiO_2_ nanoparticle‐impregnated membranes. This examination focused on assessing the membrane surface morphology, particularly the impact of adding TiO_2_ nanoparticles. The inspection was conducted in contact mode using a silicone tip (model AA3000) from Angstrom Advanced Inc., USA, at the Department of Applied Science, Baghdad University, Baghdad, Iraq.

#### 2.3.5 Contact Angle

Sessile drops were utilized to evaluate the hydrophilicity of the membrane surfaces. The contact angle between the membrane surface and distilled water was measured using a contact angle analyzer (CAM 110‐O4W, Taiwan). Each membrane was tested three times, and the average of these three readings was used for analysis. This analysis was conducted at the Department of Chemical Engineering, University of Technology, Baghdad, Iraq.

#### 2.3.6 Porosity

The membranes were first weighed when they dried, which represents Wd. The dried samples were soaked in distilled water for 24 h and then wiped to remove any remaining water from the surface. The samples were reweighed, and the measured weight represents Ww. The inspection was conducted at the Department of Materials Engineering/University of Technology, Baghdad/Iraq.

The gravimetric method was used for evaluating the porosity of membranes using the equation below (Ayaz et al. 2019):

(1) $$\varepsilon \;\left(\%\right)=\frac{\mathrm{Ww}-\mathrm{Wd}}{A\times L\times \mathrm{\rho w}}\times 100$$

where Ww is the membrane's weight when wet (g), Wd is the membrane's weight when dry (g), A is the membrane's area (cm^2^), L is the membrane's thickness (cm), and ρw is the water density (0.998 g/cm^3^).

### 2.4 Membrane Performance

#### 2.4.1 Pure Water Flux

A manually assembled crossflow (CF) filtration system was utilized to evaluate the performance of the membranes. The testing was conducted using a CF acrylic cell, along with two pressure gauges, a flow meter, a feed tank, and a pump (model no. AQ‐75GPD). Deionized water was used throughout all experiments, which were carried out at a pressure of 1 bar and at room temperature. The following equation was employed to calculate the flow of pure water (Ayaz et al. 2019):

(2) $$J=\frac{W}{t\times A}$$

where J is the pure water flux of membrane (kg/h·m^2^), W is the permeate flux weight (kg), t is the time (h), and A is the membrane area (m^2^).

#### 2.4.2 Membrane Rejection Against Dye

Membrane rejection was evaluated using a (50 ppm) rhodamine B solution as artificial wastewater. Using (UV‐Vis) spectroscopy, model (UV‐1100), at wavelength (560 nm), the final concentrations of the dye solution were determined. The following equation was used to determine the membranes' rejection (Ayaz et al. 2019):

(3) $$\text{Rejection}\;\left(\%\right)=\frac{\mathrm{Cf}-\mathrm{Cp}}{\mathrm{Cf}}\times 100$$

where C_f_ is the feed concentration (mg/L) and C_p_ is the permeate concentration (mg/L).

#### 2.4.3 Fouling Characteristics

The antifouling characteristics of both pristine and modified TiO_2_‐polymer membranes were evaluated and compared. To assess the antifouling capabilities of the membranes, BSA at a concentration of 1000 ppm was utilized. The measurements were taken at a wavelength of 280 nm, and the following parameters were calculated: FRR, irreversible flux decline ratio (R_ir_), reversible flux decline ratio (R_r_), and total flux decline ratio (R_t_). These parameters were calculated using the equations shown below (Al‐Timimi et al. 2022):

(4) $$\mathrm{FRR}\%=\left[\frac{J2}{J1}\right]\times 100\%$$

(5) $$R_{\mathrm{ir}}\%=\left[\frac{J1-J2}{J1}\right]\times 100\%$$

(6) $$R_{r}\%=\left[\frac{J2-\mathrm{Jp}}{J1}\right]\times 100\%$$

(7) $$R_{t}\%=\left[\frac{J1-\mathrm{Jp}}{J1}\right]\times 100\%$$

where J_1_ is the pure water flux before the fouling test (Lm^−2^ h^−1^), J_2_ is the cleaned membrane (pure water flux after the fouling test) (Lm^−2^ h^−1^), and J_p_ is the pure water flux of the BSA solution (Lm^−2^ h^−1^).

## 3 Results and Discussion

### 3.1 XRD

**FIGURE 2:**
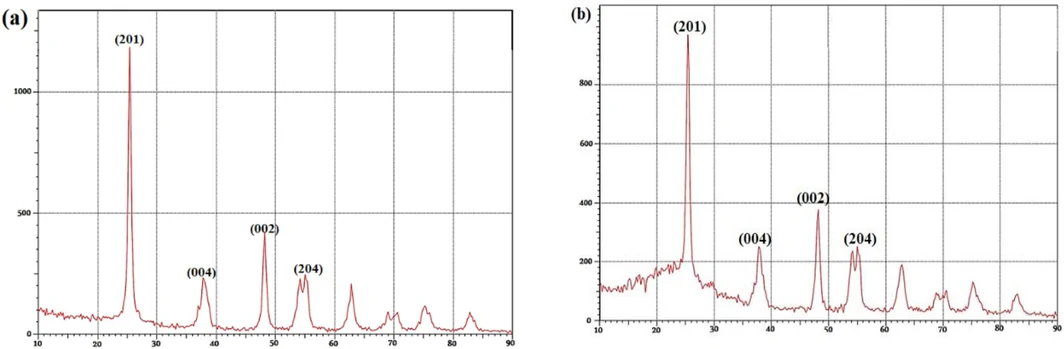
(a) XRD of anatase TiO_2_. (b) XRD of H_3_PO_4_‐modified TiO_2_.

Figure 2 reveals XRD of anatase TiO_2_ and H_3_PO_4_‐modified TiO_2_. The unmodified TiO_2_ pattern at (2θ) of crystal powders exhibits crystalline characteristic peaks of 25.1°, 38.6°, 48.4°, and 56.1°, which can be indexed with (201), (004), (002), and (204) values of Miller indices of anatase TiO_2_ crystal faces (Figure 2a). These results were in good agreement with the Joint Committee on Powder Diffraction Standards (JCPDS) card number (21‐1272) (Wang et al. 2020). The crystalline characteristic peaks in the H_3_PO_4_‐modified TiO_2_ pattern of (2θ) of crystal powders showed a slight shift in peak locations when compared to unmodified TiO_2_, as shown in Figure 2b. This shift was attributed to the incorporation of TiO_2_ and phosphoric acid, in which Ti_4_^+^ was replaced by a pentavalent (P_5_^+^) cation, shifting the energy bands to enable rapid photoexcitation and flow at a lower energy level (Fan et al. 2008).

### 3.2 FTIR

**FIGURE 3:**
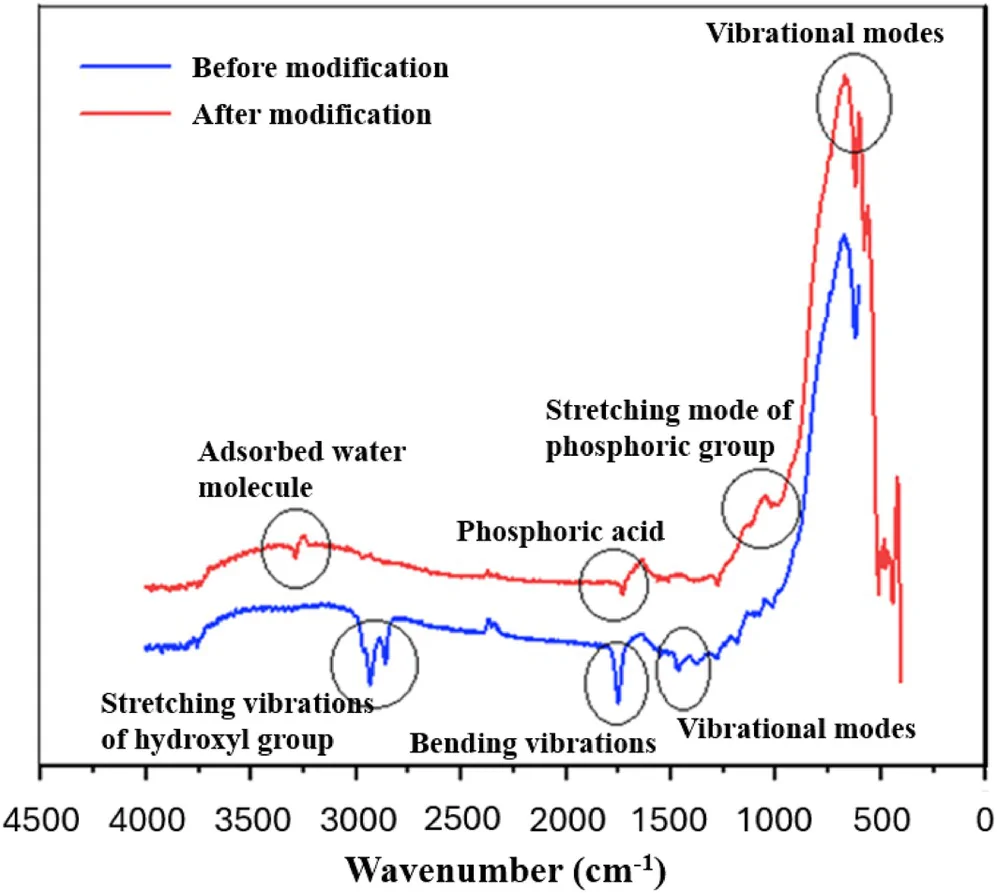
FTIR spectroscopy of unmodified TiO_2_ and H_3_PO_4_‐modified TiO_2_ nanoparticles.

Figure 3 compares FTIR spectra of unmodified TiO_2_ nanoparticles and H_3_PO_4_‐modified TiO_2_. With the aid of FTIR spectroscopy, the spectrum of unmodified TiO_2_ nanoparticles shows several peaks at 2882.50, 1656.90, and ≈1405.37 cm^−1^ and an intense broad band in the range of 400–850 cm^−1^. The peak seen at 2882.50 cm^−1^ is due to stretching vibrations of OH (Debnath et al. 2019), whereas the peak at 1656.90 cm^−1^ is attributed to the stretching vibrations of the Ti–OH bond (Sathish and Madhvesh 2023). The presence of OH bands in the spectrum is due to adsorbed H_2_O on the nanoparticles' surfaces. The band at 1405.37 cm^−1^ is assigned to the Ti–O–Ti vibration (Saravanan et al. 2010). The intense broad band in the range of 400–850 cm^−1^ could be attributed to Ti–O and Ti–O–Ti bonds originating from the native oxide layer of the titanium dioxide network of the film (Sathish and Madhvesh 2023).

FTIR spectroscopy of H_3_PO_4_‐modified TiO_2_ nanoparticles revealed several key peaks. A peak around 3400 cm^−1^ was attributed to OH stretching vibrations, whereas a peak at 1630 cm^−1^ corresponded to O–H–O bending vibrations (Mojet et al. 2010). Additionally, the peak at approximately 1750 cm^−1^ was associated with the scissoring vibration of the terminal OH group (Tarakanova et al. 2024). Notably, a new peak appeared in the 1000–1200 cm^−1^ range, corresponding to the symmetric stretching mode of the phosphate group.

#### 3.2.1 FTIR of Modified TiO_2_‐PES Membranes

**FIGURE 4:**
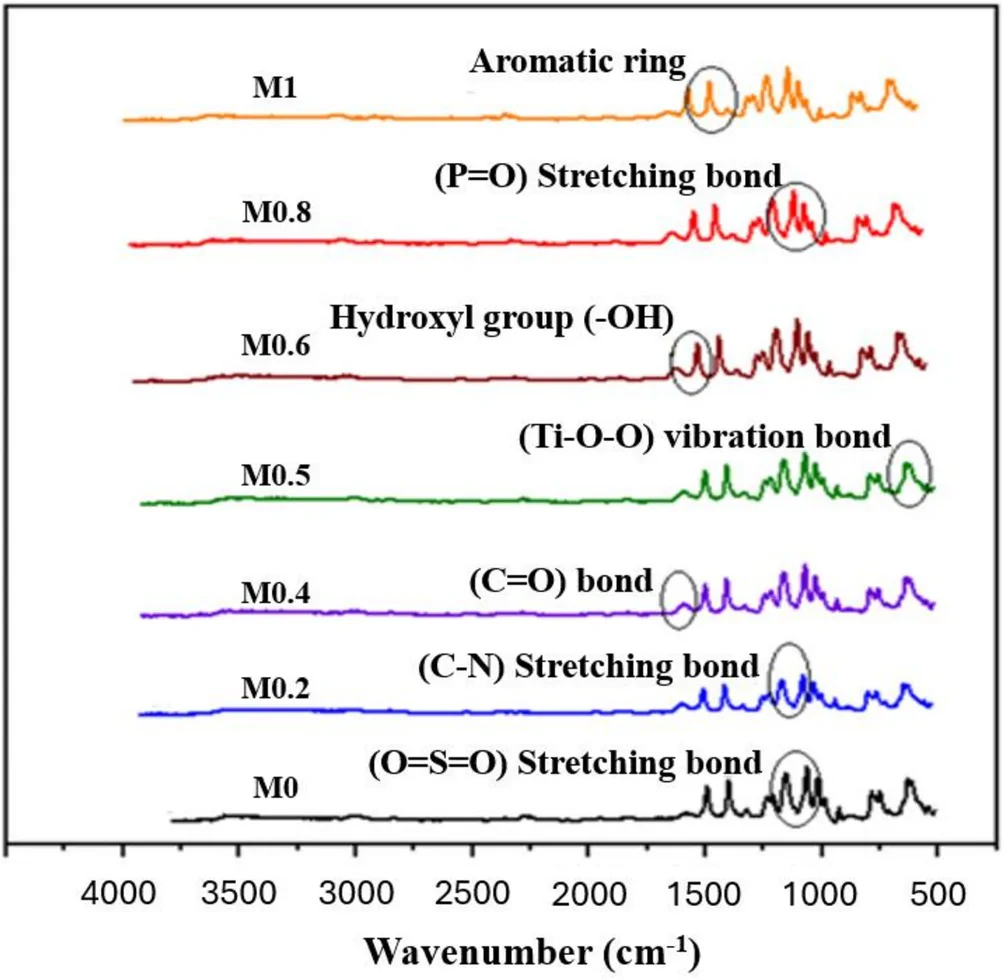
FTIR of neat PES and H_3_PO_4_‐modified TiO_2_‐PES membranes.

Figure 4 displays the infrared absorption spectra for the samples (M_0_, M_0.2_, M_0.4_, M_0.5_, M_0.6_, M_0.8_, and M_1_). The peak at 1149.58 cm^−1^ is attributed to the stretching of the (O=S=O) bond (Fashedemi et al. 2015), whereas the peak at 1103.94 cm^−1^ corresponds to the (C–O) stretching bond (Smith 2017). Additionally, the peak at 1239.13 cm^−1^ is ascribed to the (C–O–C) bond (Hu et al. 2019). Peaks at 1485.53 and 1577.57 cm^−1^ are associated with the aromatic ring, whereas a peak represents the C–H stretching at 3097 cm^−1^ (Smith 2016).

Furthermore, the C–N stretching of PVP appears as a peak at 1296.81 cm^−1^ (Rahma et al. 2016), and the peak at 1657.66 cm^−1^ is linked to the C=O bond (Ahmed and Abdullah 2020). A peak at 3301.98 cm^−1^ corresponds to the O–H stretching (Md Salim et al. 2021). Another peak around 590 cm^−1^ is due to the Ti–O–O vibration bond (Vasconcelos et al. 2011). This peak appears in the same frequency regime as the C–S group in the pristine membrane. Surface hydroxyl groups (−OH) are indicated by a peak at 1630 cm^−1^, and adsorbed water molecules produce a peak at 3600 cm^−1^ (Vasconcelos et al. 2011; Kitadai et al. 2014; Zhou et al. 2023). The peak at 1750 cm^−1^ appears for the C=O bonds (Al Lafi 2014). Notably, the strong peak between ≈990–1100 cm^−1^ represents the phosphate group (PO_4_^3−^) as asymmetric stretching mode of the P–O and/or P=O bond (Withnall and Andrews 1987; Jastrzębski et al. 2011), which is indicative of phosphoric acid modifying the TiO_2_.

### 3.3 SEM and EDS Analysis for the Modified TiO_2_‐PES Membranes

**FIGURE 5:**
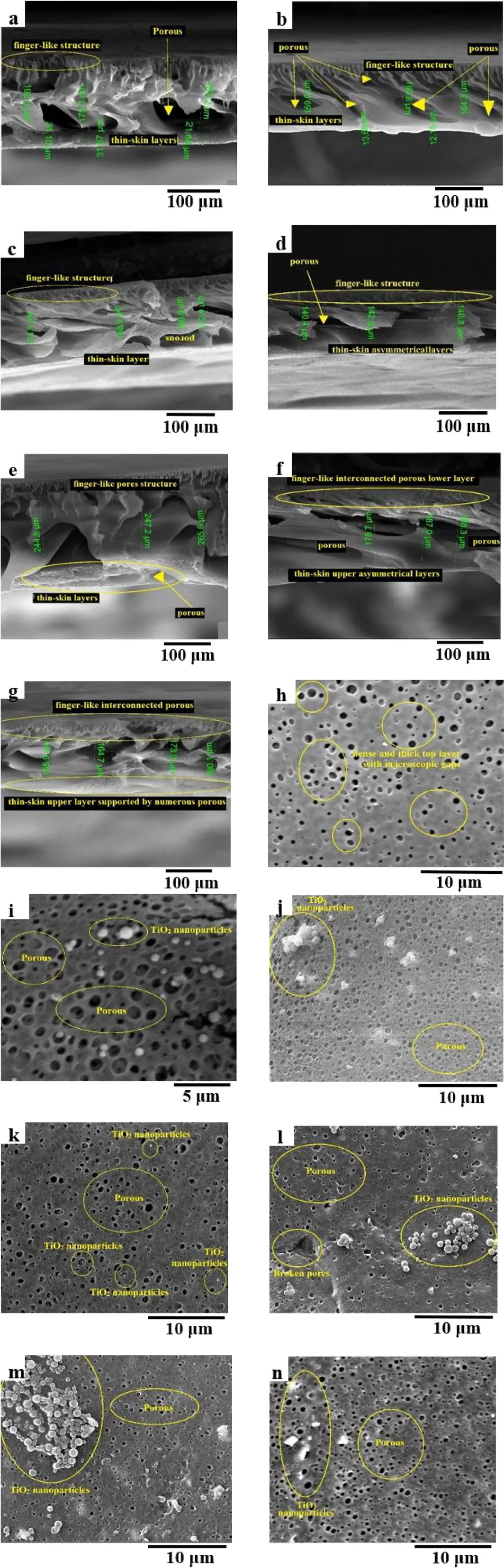
Cross‐sectional image ((a) M_0_, (b) M_0.2_, (c) M_0.4_, (d) M_0.5_, (e) M_0.6_, (f) M_0.8_, and (g) M_1_) and top surface image ((h) M_0_, (i) M_0.2_, (j) M_0.4_, (k) M_0.5_, (l) M_0.6_, (m) M_0.8_, and (n) M_1_).

Figure 5a–g illustrates micrographs of PES membranes' cross‐sections. All membranes displayed asymmetrical structures, including thin‐skin layers supported by numerous pores with minor variations. Due to the incorporation of the polymeric membrane at the microscale, the skin layer protruded into a finger‐like structure in the upper half of the membrane. With the introduction of TiO_2_, the creation of finger‐like pores increased due to the hydrophilic characteristic of TiO_2_. The finger‐like, interconnected porous architecture is clearly observed throughout the membrane thickness, enhancing permeability and reducing mass transfer resistance.

The top surface morphologies of (M_0_, M_0.2_, M_0.4_, M_0.5_, M_0.6_, M_0.8_, and M_1_) membranes are illustrated in Figure 5h–n. The membrane of pure PES exhibits a surface morphology characterized by a dense and thick top layer with macroscopic gaps. Typically, the membrane of pure PES has a non‐symmetric shape, featuring a dense, thin top layer filled with locked cells within a polymer matrix and a porous sub‐layer underneath. The surface morphology of the modified TiO_2_‐PES membranes showed a highly porous surface and a visible sponge‐like cross‐section. A sufficient amount of TiO_2_ nanoparticles were uniformly embedded on the outer and pore surfaces of the membranes. Although the membrane's structure did not change with increasing TiO_2_ concentration, raising the TiO_2_ ratio generated a large number of collapses, also causing small aggregation in the membranes and creating broken pores, thereby increasing the closeness of TiO_2_ aggregates at the surface and cross‐section of the membranes (specifically M_0.6_, M_0.8_, and M_1_ as shown in Figure 5l–n).

The EDS examination of the modified TiO_2_‐PES membranes provides important information about the samples' elemental composition, as the titanium content varies from 0% to 1% in the prepared membranes (Figure S2a–g). For M_0_, the baseline sample contains no titanium and is composed mainly of other elements, such as carbon at a high percentage, followed by oxygen and sulfur. As the percentage of titanium goes up from 0.2 to 1 (i.e., for the membranes M_0.2_–M_1_), the height of the titanium peak in the EDS spectrum also goes up. The fact that the titanium peak has increased is a clear indication that the modified TiO_2_ particles are being successfully impregnated into the membrane's structure. By reaching a 1% concentration of modified nanoparticles in the M_1_ membrane structure, the titanium peak is significantly higher, indicating that the membrane now contains a substantial amount of titanium. Other elements such as carbon, oxygen, sulfur, and phosphorus are detected in membrane samples M_0.2_–M_1_. The carbon peak remains relatively stable across all samples, indicating that carbon is a consistent component of the membrane's composition, presumably originating from the polymer matrix. Oxygen, another important element, is likely included because it is present in the polymer matrix, and titanium dioxide (TiO_2_) naturally contains oxygen. The sulfur and phosphorus peaks suggest that these elements are in the membrane material. Table 2 summarizes the atomic and weight percentages of the elements in each membrane sample.

**TABLE 2:** Elemental composition of modified TiO_2_‐PES membranes at varying titanium concentrations (atomic and weight percentages).

Membrane code	Elements (atomic %)					Elements (weight %)				
	Ti	C	O	S	P	Ti	C	O	S	P
M_0_		64.5	29.6	5.9			53.9	33.0	13.1	
M_0.2_	0.3	66.0	28.0	5.6	0.1	0.9	55.2	31.0	12.6	0.3
M_0.4_	0.4	64.4	29.7	5.4	0.1	1.1	53.7	32.9	12	0.3
M_0.5_	0.8	65.4	26.3	7.4	0.1	2.6	52.7	29.2	15.2	0.3
M_0.6_	0.6	65.4	28.1	5.8	0.1	2.1	53.6	31.0	12.8	0.5
M_0.8_	0.9	63.2	30.0	5.8	0.1	2.9	51.6	32.4	12.6	0.5
M_1_	1.1	64	29	5.8	0.1	3.7	51.6	31.5	12.7	0.5

### 3.4 AFM of the Modified TiO_2_‐PES Membranes

**FIGURE 6:**
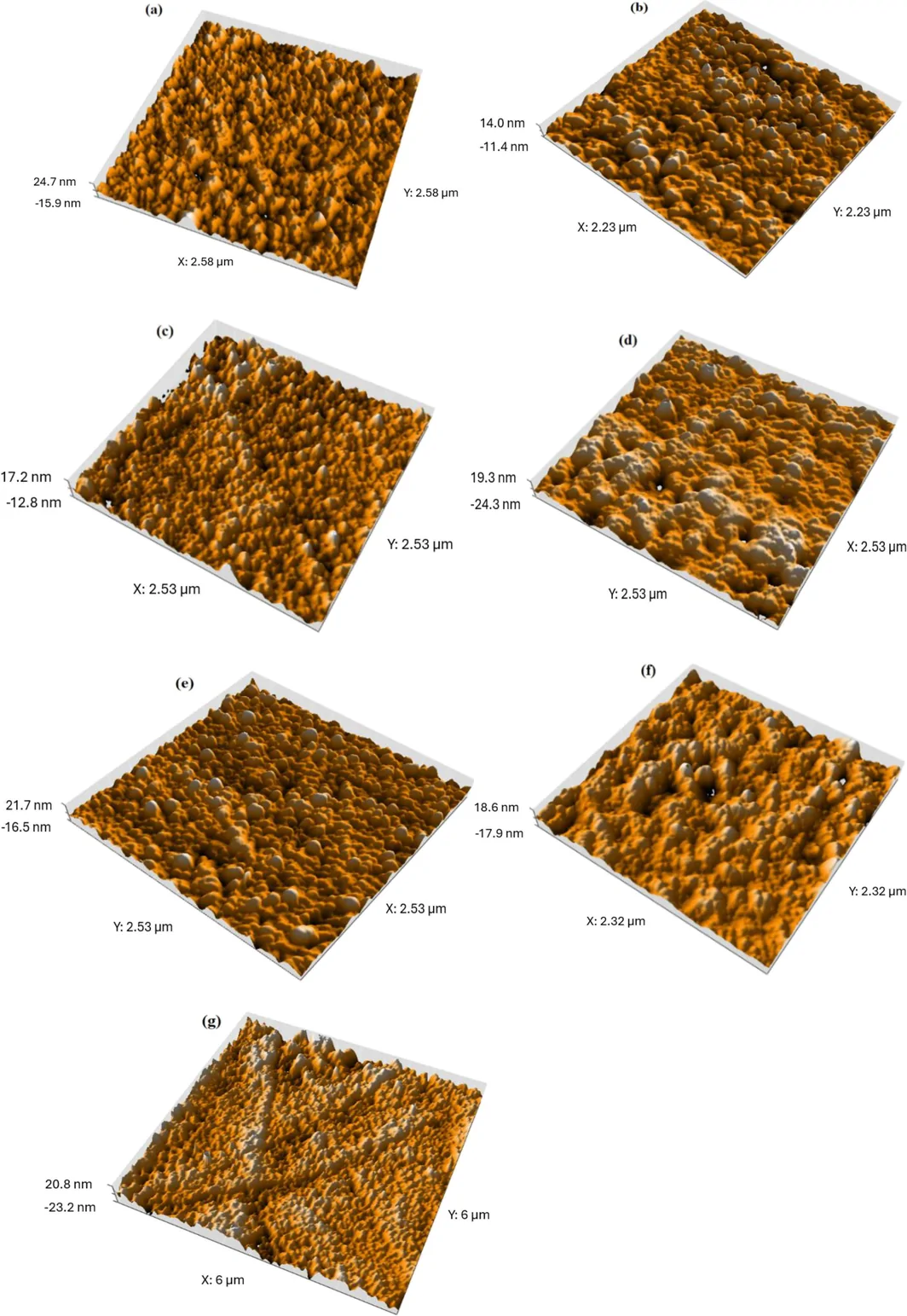
AFM images (a) M_0_, (b) M_0.2_, (c) M_0.4_, (d) M_0.5_, (e) M_0.6_, (f) M_0.8_, and (g) M_1_.

The AFM technique was used to investigate the 3D surface roughness and morphology of the neat and composite membranes, as shown in Figure 6 for membranes with and without modified TiO_2_ nanoparticles. The roughness of the top and bottom surfaces of PES membranes increased with increasing TiO_2_ nanoparticle content from 0 to 1 wt%. The results are attributed to TiO_2_'s hydrophilic nature, which, during the phase‐inversion manufacturing process, facilitates rapid phase exchange and allows nanopores to diffuse across the membrane from the top to the bottom surfaces. The AFM image showed light colors representing the peaks and dark colors representing the lowest areas (valleys) of the membranes.

Results showed that the membranes modified with (0.5 wt%) TiO_2_ for PES decreased in the mean roughness values (Sa), and the values increased beyond this value of TiO_2_ percentage. The unmodified membrane (M_0_) showed a higher mean surface roughness (5.645 nm) than the treated membranes with modified TiO_2_ nanoparticles. It is well known that the fouling tendency decreases with increasing membrane surface smoothness (Shah et al. 2019; Wu et al. 2019).

Table 3 shows the values of the mean roughness (Sa) of the membranes as well as the mean pore size (nm), thus determined from the AFM measurements by particle analysis‐ threshold detection. The results indicated a reduction in surface roughness for the modified membranes when the nano‐additive ratio increased to 0.2 wt% and 0.4 wt%. Compared to the membrane without TiO_2_, the enhancement in the topography of membranes impregnated with the modified TiO_2_ nanoparticles is attributed to the entrapping of nanoparticles within the polymer matrix. However, above 0.4 wt% TiO_2_, the roughness increased, consistent with SEM results showing nanoparticle aggregation at the top of the polymeric matrix of the developed membranes. Previous studies reported that when TiO_2_ particles are added in excess to the matrix, bumps form, thereby increasing the peak value (Wu et al. 2019; Birsan et al. 2021; Yanuhar et al. 2022).

**TABLE 3:** Mean roughness and mean pore size of PES membranes.

Membrane code	TiO_2_ (wt%)	Mean roughness (Sa) (nm)	Mean pore size (m) (nm)
M_0_	—	5.645	57.49
M_0.2_	0.2	4.361	35.48
M_0.4_	0.4	4.381	41.62
M_0.5_	0.5	5.954	43.28
M_0.6_	0.6	5.823	42.96
M_0.8_	0.8	5.498	50.50
M_1_	1	5.317	50.50

### 3.5 Contact Angle for the Modified TiO_2_‐PES Membranes

**FIGURE 7:**
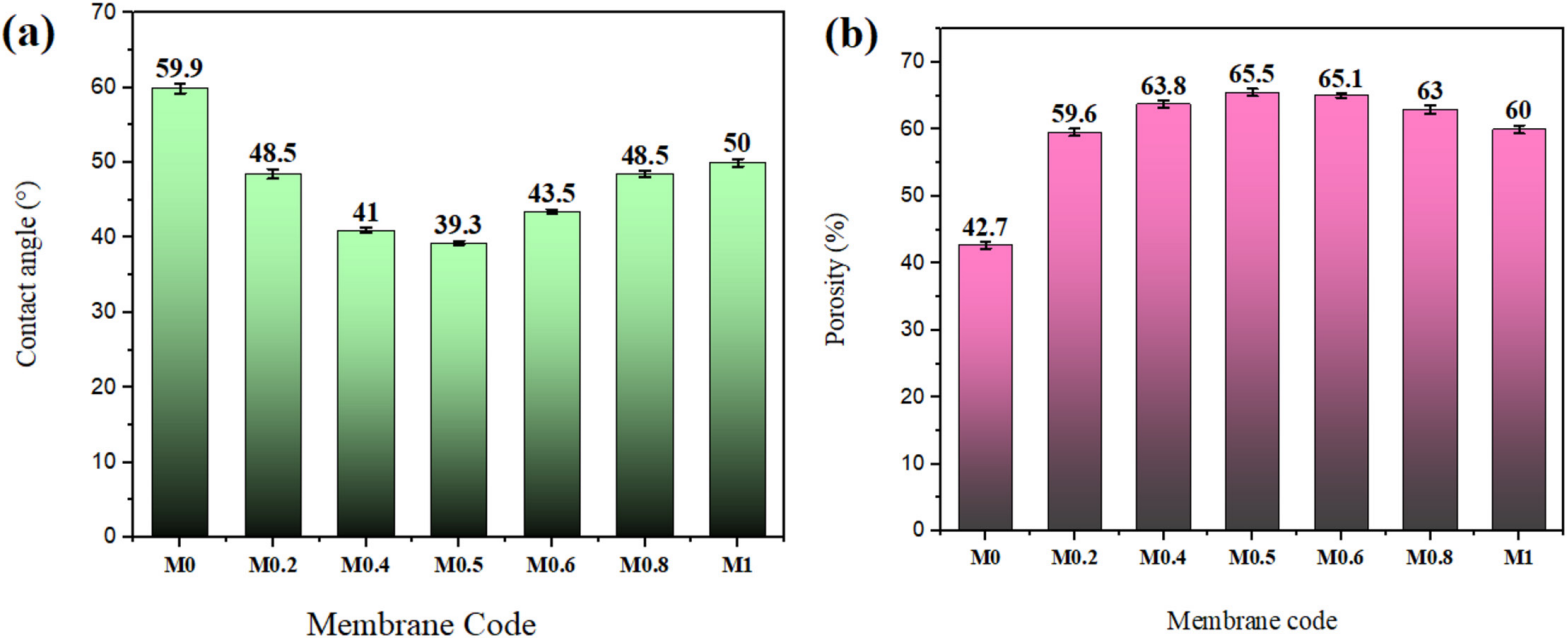
(a) Contact angle and (b) porosity of the modified TiO_2_‐PES membranes.

Figure 7a shows the hydrophilicity of neat PES and H_3_PO_4_‐modified TiO_2_‐PES membranes, which were examined by contact angle. The data of contact angle of neat PES and the modified membranes with different concentrations of TiO_2_ nanoparticles (0.2, 0.4, 0.5, 0.6, 0.8, and 1 wt%). This study's results indicated that the addition of TiO_2_ nanoparticles increased the membranes' hydrophilicity, attributed to the nanoparticles' presence. The contact angle value of the neat PES membrane showed about (59.9°), and results showed that there was a slight, gradual decrease in the value due to the addition of the modified nanoparticles. The contact angle of the membranes decreased with an increasing ratio of TiO_2_, and then it reached 39.3° for the M_0.5_ membrane that was prepared with 0.5 wt% TiO_2_, which is the optimal contact angle value. This improvement in hydrophilicity was caused by hydrophilic TiO_2_ nanoparticles migrating to the membrane surface during phase inversion, thereby decreasing interfacial energy and increasing surface hydrophilicity. Scrutiny of Figure 7a reveals that the contact angle values increased slightly as more TiO_2_ was added, reaching 50° at 1 wt% of TiO_2_, which is still lower than that of the membrane without TiO_2_ nanoparticles. Generally, the results showed that the addition of TiO_2_ nanoparticles increased the membrane's hydrophilicity, resulting in a decrease in the contact angle of the modified membranes (viz., as the TiO_2_ content increased, the contact angle decreased, indicating an increase in the membrane's hydrophilicity).

### 3.6 Porosity for the Modified TiO_2_‐PES Membranes

Figure 7b shows the membrane porosity of neat PES membranes (i.e., without TiO_2_ nanoparticles) and PES membranes impregnated with the modified TiO_2_ nanoparticles at different concentrations. The results showed that the PES membrane without TiO_2_ nanoparticles had a porosity of 42.7%. However, with the addition of TiO_2_ nanoparticles, the increase in porosity continued gradually, reaching 65.5% for the membrane impregnated with 0.5% TiO_2_ nanoparticles (M_0.5_). Then the porosity of membranes began to decrease and reached (60%) at 1 wt% TiO_2_, and the reduction in porosity of membranes by increasing the nanoparticles content more than 0.5 wt% was attributed to the higher concentration of TiO_2_ nanoparticles, which led to a decrease in permeability because of pore blockage caused by excessive TiO_2_.

### 3.7 Membrane Performance for the Modified TiO_2_‐PES Membranes

#### 3.7.1 Pure Water Flux for the Modified TiO_2_‐PES Membranes

**FIGURE 8:**
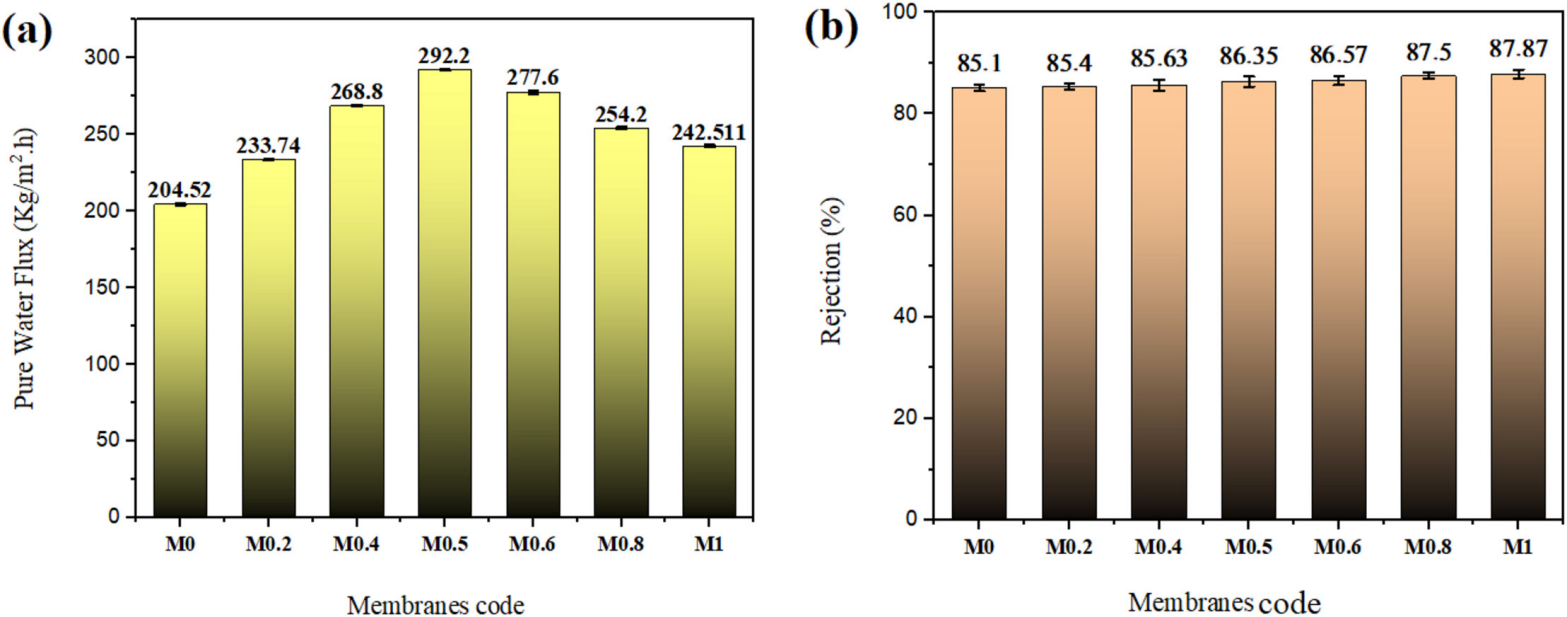
(a) Pure water flux and (b) rejection for the PES membranes impregnated with TiO_2_ nanoparticles.

Figure 8a demonstrates that the inclusion of TiO_2_‐H_3_PO_4_ modified nanoparticles in PES membranes significantly enhanced the pure water flux. The results indicated that the flux for the membrane without nanoparticles (M_0_) was 204.52 L/m^2^ h. In contrast, the addition of modified TiO_2_ nanoparticles increased the pure water flux to 292.18 L/m^2^ h for the M_0.5_ membrane, which recorded the highest value. It was evident that the pure water flux through the membrane improved noticeably when the nanoparticle content reached 0.5 wt%. This enhancement can be attributed to improvements in various characteristics, such as porosity, pore size, and hydrophilicity. However, the results also showed that increasing the TiO_2_ content beyond 0.5 wt% in the PES membrane resulted in a significant increase in water rejection. This decrease in water flux results from reduced total porosity due to pore closure at higher nanoparticle concentrations.

#### 3.7.2 Membrane Rejection Against Dye for the Modified TiO_2_‐PES Membranes

Figure 8b shows the membrane's dye rejection for Rhodamine B dye for (M_0_, M_0.2_, M_0.4_, M_0.5_, M_0.6_, M_0.8_, and M_1_) membranes. As demonstrated in the results, both the pristine PES membrane and the PES membranes impregnated with the modified TiO_2_ nanoparticles show an increase in dye rejection. For instance, results indicated that the pristine PES membrane exhibited rejection of rhodamine B at 85.1%, whereas the highest rejection was 87.87% for the M_1_ membrane at 1 wt% TiO_2_ nanoparticle concentration. Several factors may contribute to the increase in rejection, including internal pore narrowing, the formation of a dense gel layer on the membrane surface, and pore clogging.

Rhodamine B, a cationic dye with a molecular weight of 479 g/mol in its chloride form, contains an ammonium group that can bond electrostatically to phosphate groups. The longest dimension of the rhodamine B molecule is reported to be 1.77 nm (Canning et al. 2014). Because the size of the ammonium groups and phosphate groups in the functionalized membrane is smaller than the pores on its surface, the primary mechanism for dye adsorption is electrostatic binding rather than sieving. In diluted aqueous solutions, PES membranes exhibit a slight negative charge, resulting in an attraction between the dye molecules and the membrane due to the charge difference (Ghiasi et al. 2019). This interaction enhances the membranes' rejection capability under both sunlight and UV light.

Furthermore, the pH of the water solution has a significant effect on the charge interactions, which is crucial. When the pH is low, the dye molecules and the membrane may have different charges. The phosphate groups on the membrane surface can be negatively charged in acidic conditions. This may make them interact more strongly with the positively charged ammonium groups of rhodamine B. This electrostatic attraction can make it more difficult for the dye to pass through, especially when the charges on the membrane and dye are opposing. However, the TiO_2_ nanoparticles on the membrane surface can alter both the surface charge and the total electrostatic interactions, even though high TiO_2_ concentrations can cause pore collapse.

#### 3.7.3 FRR and Fouling Resistance for the Modified TiO_2_‐PES Membranes

**FIGURE 9:**
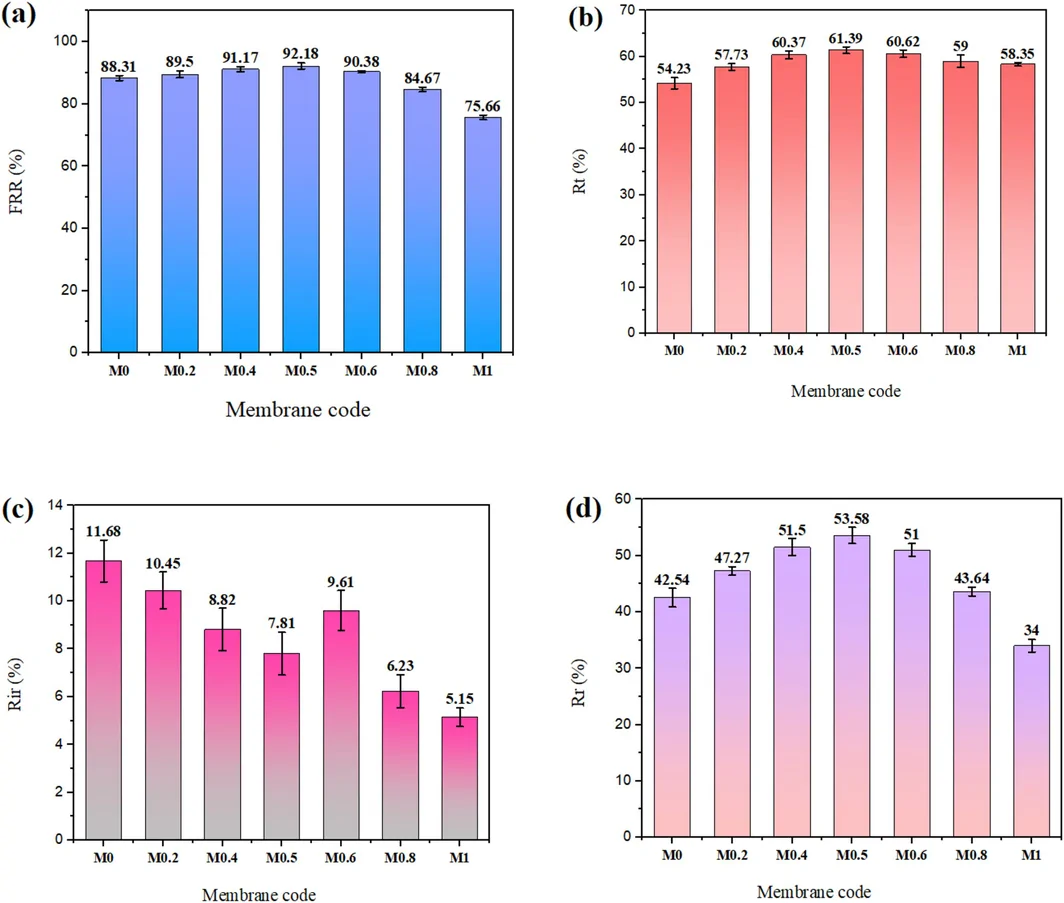
(a) Flux recovery ratio, (b) total flux decline ratio (R_t_) of the membranes, (c) irreversible flux decline ratio (R_ir_) of the membranes, and (d) reversible flux decline ratio (R_r_) of the membranes.

Figure 9a illustrates the FRR, confirming the effectiveness of the modified membranes. The FRR is frequently used for measuring membrane fouling resistance. For 2 h at 1 bar, all membranes were backwashed with distilled water. Results indicated that after backwashing, the pure water flux was lower than before, suggesting that contaminants remained on the membrane's surface. Results showed a noticeable difference between the values of all membranes, including pristine PES membranes and those with modified nanoparticles. The maximum FRR was related to M_0.5_ with a value (92.18%) compared with 88.31%, 89.5%, 92.17%, 90.38%, 84.67%, and 75.66% for M_0_, M_0.2_, M_0.4_, M_0.6_, M_0.8_, and M_1_ membranes, respectively. The higher flux was observed for the membrane impregnated with 0.5% of the modified TiO_2_ nanoparticles (M0.5) due to its hydrophilic properties, which led to weaker adsorption of pollutants on the membrane surface compared to the membrane with the highest amount of nanoparticles (M_1_). Fouling of the membranes is divided into reversible fouling (R_r_, which restores the permeate flux by cleaning) and irreversible fouling (R_ir_), in which the fouling exists, even after extensive cleaning. A part of the flux cannot be recovered, and Rt is the membrane total flux decline ratio. Results show that all values of total flux decline ratio (Rt) for composite membranes were higher than those of the PES membranes without nanoparticles (M_0_), as shown in Figure 9b. The R_t_ lower values of M_0_ membrane indicate that the membranes with modified TiO_2_ have enhanced characteristics and long‐term stability against fouling. The maximum Rt value was related to M_0.5_ with a value (61.39%) compared to 54.23%, 57.73%, 60.37%, 60.62%, 59%, and 58.35% for M_0_, M_0.2_, M_0.4_, M_0.6_, M_0.8_, and M_1_ membranes, respectively.

Figure 9c shows that the irreversible fouling (R_ir_) values decreased for all membranes containing modified TiO_2_ nanoparticles compared to the pristine PES membrane without nanoparticles. For instance, the R_ir_ for the pristine PES membrane was more than one and a half times higher than that of the membrane impregnated with 0.5% TiO_2_ nanoparticles. When the nanoparticle concentration was doubled to 1% in the M_1_ membrane, the R_ir_ decreased to 5.15%, compared to 11.68% for the pristine membrane (M_0_). However, the reversible fouling (R_r_) values of membranes, shown in Figure 9d, increased with the addition of TiO_2_ nanoparticles to PES membranes. Thus, the results show that the R_r_ of (M_0_) was 42.54% and increased with the addition of modified TiO_2_ nanoparticles to membranes, reaching 53.58% for the M_0.5_ membrane. Increasing Rr values with increased TiO_2_ nanoparticle concentration demonstrate the best self‐cleaning capability. However, there is a slight decrease in Rr as the percentage of nanoparticles increases above 0.5%, reaching 43.64% for the membrane with 0.8% TiO_2_ nanoparticles. Lower Rr values indicate greater antifouling and suggest that rhodamine B adsorbed on the membranes is easily washable. Herein, the smallest Rr values indicate that most of the flux could be recovered with minimal cleanup. Table S1 compares membrane performance parameters for membranes with and without acid‐modified TiO_2_ nanoparticles.

## 4 Conclusions

In this study, PES ultrafiltration membranes were successfully modified by incorporating phosphoric acid–modified TiO_2_ nanoparticles at varying loadings to enhance membrane hydrophilicity, permeability, antifouling properties, and dye‐removal performance for wastewater treatment applications. Structural and chemical characterizations (XRD and FTIR) confirmed the successful surface modification of TiO_2_ nanoparticles and their effective incorporation into the PES matrix. At the same time, SEM, EDX, and AFM analyses revealed a well‐developed asymmetric membrane structure with improved pore architecture at optimal nanoparticle content.

The addition of modified TiO_2_ significantly improved membrane surface properties, as evidenced by a substantial reduction in water contact angle and an increase in porosity. Among all prepared membranes, the membrane containing 0.5 wt% modified TiO_2_ (M0.5) demonstrated the most balanced and superior performance. This membrane exhibited the lowest contact angle (≈39°), the highest porosity (≈65.5%), and the highest pure water flux (≈292 L·m^−2^·h^−1^), representing a significant improvement over the pristine PES membrane. These improvements are attributed to the hydrophilic nature of the phosphate‐functionalized TiO_2_ nanoparticles.

In terms of separation performance, all TiO_2_‐modified membranes showed improved rejection of rhodamine B dye compared to the pristine PES membrane, with rejection values exceeding 85%. The enhancement in dye removal was governed by a combination of electrostatic interactions between phosphate groups on the membrane surface and the cationic dye molecules, as well as pore structure modification. Although higher TiO_2_ loadings slightly increased dye rejection, excessive nanoparticle content (> 0.5 wt%) led to pore blockage and partial nanoparticle agglomeration, resulting in reduced permeability and less favorable antifouling behavior.

Fouling resistance evaluations using BSA as a model foulant demonstrated that TiO_2_ incorporation markedly improved membrane antifouling properties. The M_0.5_ membrane achieved the highest flux recovery ratio (≈92%), the lowest irreversible fouling resistance, and enhanced reversible fouling behavior, confirming that membrane fouling was predominantly reversible and easily mitigated by simple hydraulic cleaning. These results highlight the crucial role of optimized nanoparticle loading in achieving long‐term membrane stability and operational efficiency.

Despite the promising results, several limitations should be acknowledged. Membrane performance was evaluated using rhodamine B and BSA model solutions under controlled conditions; therefore, further studies using real industrial wastewater are required. In addition, long‐term stability, nanoparticle leaching, and membrane durability were not comprehensively investigated. Future work should focus on long‐term operation under realistic wastewater treatment conditions, including fouling behavior and cleaning efficiency. Further studies on membrane–foulant interactions, large‐scale applicability, and the removal of emerging contaminants, pharmaceuticals, and heavy metals are also recommended.

## Author Contributions

**Assala M. Al‐Jubouri:** conceptualization, investigation, methodology, validation, writing – review and editing. **Kadhum M. Shabeeb:** conceptualization, writing – review and editing, validation. **Qusay F. Alsalhy:** conceptualization, methodology, validation, writing – review and editing, formal analysis, supervision, investigation. **Saad Al‐Saadi:** conceptualization, methodology, formal analysis, supervision, writing – review and editing, investigation. **Adel Zrille:** validation, formal analysis, writing – review and editing. **Muayad Al‐shaeli:** validation, formal analysis, writing – review and editing.

## Funding

The authors wish to acknowledge the financial support provided by the Ministry of Higher Education and Scientific Research in Iraq.

## Conflicts of Interest

The authors declare no conflicts of interest.

## Supporting information


**Figure S1:** Membrane preparation process and the interaction mechanism between polyethersulfone (PES) and titanium oxide (TiO_2_) nanoparticles during the membrane fabrication occurs at the molecular level.
**Figure S2:** EDS of membranes (a) M_0_, (b) M_0.2_, (c) M_0.4_, (d) M_0.5_, (e) M_0.6_, (f) M_0.8_, and (g) M_1_.
**Table S1:** Membranes' physicochemical and filtration performance parameters (contact angle, porosity, pure water flux [PWF], rejection, flux recovery ratio [FRR], total flux decline ratio [R_t_], irreversible flux decline ratio [R_ir_], and reversible flux decline ratio [R_r_]).

## Data Availability

Data are contained within the article.
