# Supplementary material for: Phosphoric Acid–Modified TiO2 Nanoparticles as Functional Additives for High‐Performance Polyethersulfone Ultrafiltration Membranes
Source: Water Environ Res. 2026 Sep 10;98(9):e70576. doi: 10.1002/wer.70576 (PMC13562761; doi:10.1002/wer.70576)
Supplement: Supplementary file 1 [file WER-98-e70576-s001.docx]

Phosphoric Acid-Modified TiO_2_ Nanoparticles as Functional Additives for High Performance Polyethersulfone Ultrafiltration Membranes

Assala M. Al-Jubouri^1^, Kadhum M. Shabeeb^1^, Qusay F. Alsalhy^*2^, Saad Al-Saadi^*3^, Adel Zrille^4^, Muayad al-shaeli^5^

^1^Materials Engineering Department, University of Technology-Iraq, Alsinaa Street 52, 10066 Baghdad, Iraq

^2^Membrane Technology Research Unit, Chemical Engineering Department, University of Technology-Iraq, Alsinaa Street 52, 10066 Baghdad, Iraq

^3^Department of Mechanical and Aerospace Engineering, Monash University, Clayton, Victoria 3800, Australia

^4^Research Unit Advanced Materials, Applied Mechanics, Innovative Processes and Environment, Higher Institute of Applied Sciences and Technology of Gabes (ISSAT), University of Gabes, 6029 Gabes, Tunisia

^5^Institute for Sustainability and Innovation, Victoria University, P.O. Box 14428, Melbourne, Victoria 8001, Australia

*****	Corresponding authors: Saad Al-Saadi ([saad.al-saadi@monash.edu](mailto:saad.al-saadi@monash.edu)), Qusay F. Alsalhy ([qusay.f.abdulhameed@uotechnology.edu.iq](mailto:qusay.f.abdulhameed@uotechnology.edu.iq)).

Supplementary document

Figure S1: Membrane preparation process and the interaction mechanism between polyethersulfone (PES) and titanium oxide (TiO_2_) nanoparticles during the membrane fabrication occurs at the molecular level.

Figure S2: EDS of membranes (a) M_0_, (b) M_0.2_, (c) M_0.4_, (d) M_0.5_, (e) M_0.6_, (f) M_0.8_ and (g) M_1_.

Table S1: Membranes physicochemical and filtration performance parameters (contact angle, porosity, pure water flux (PWF), rejection, flux recovery ratio (FRR), total flux decline ratio (R_t_), irreversible flux decline ratio (R_ir_) and reversible flux decline ratio (R_r_)).

| Membrane Code | **Contact angle (°)** | **Porosity (%)** | **PWF (Kg/m^2^.h)** | **Rejection (%)** | **FRR (%)** | **R_t_ (%)** | **R_ir_ (%)** | **R_r_ (%)** |
| --- | --- | --- | --- | --- | --- | --- | --- | --- |
| M0 | 59.9 | 42.7 | 204.52 | 85.1 | 88.31 | 54.23 | 11.68 | 42.54 |
| M0.2 | 48.5 | 59.6 | 233.74 | 85.4 | 89.5 | 57.73 | 10.45 | 47.27 |
| M0.4 | 41 | 63.8 | 268.8 | 85.63 | 91.17 | 60.37 | 8.82 | 51.5 |
| M0.5 | 39.3 | 65.5 | 292.2 | 86.35 | 92.18 | 61.39 | 7.81 | 53.58 |
| M0.6 | 43.5 | 65.1 | 277.6 | 86.57 | 90.38 | 60.62 | 9.61 | 51 |
| M0.8 | 48.5 | 63 | 254.2 | 87.5 | 84.67 | 59 | 6.23 | 43.64 |
| M1 | 50 | 60 | 242.511 | 87.87 | 75.66 | 58.35 | 5.15 | 34 |
